# Supplementary material for: A peptide triple agonist of GLP-1, neuropeptide Y1, and neuropeptide Y2 receptors promotes glycemic control and weight loss
Source: Sci Rep. 2023 Jun 12;13:9554. doi: 10.1038/s41598-023-36178-1 (PMC10261008; doi:10.1038/s41598-023-36178-1)
Supplement: Supplementary file 1 — Supplementary Information 1. [file 41598_2023_36178_MOESM1_ESM.docx]

**SUPPLEMENTARY MATERIALS;** Robert P. Doyle *et al*.,

A Peptide Triple Agonist of GLP-1, Neuropeptide Y1, and Neuropeptide Y2 Receptors Promotes Glycemic Control and Weight Loss

| REAGENT | Company/Source | Catalog # |
| --- | --- | --- |
| Antibodies | | |
| Anti-anti-Cy5 Antibody (B-2) Alexa Fluor 647 | Santa Cruz Biotechnology | sc-166896 AF647 |
| Bacterial and virus strains | | |
| H188 Adenovirus | Lab of Prof. Kees Jalink, Division of Cell Biology, the Netherlands Cancer Institute, Amsterdam, the Netherlands. | N/A |
| Biological samples | | |
| Rat Islets | Harvested from Sprague-Dawley rats (n=; ~250g; Envigo/Harlan) | N/A |
| Human Islets | Human islets were provided by the NIH-funded Integrated Islet Distribution Program | N/A |
| Rat brain tissue | Extracted from adult male Sprague-Dawley rats (n=; ~400g; Charles River Laboratories) | N/A |
| Chemicals, peptides, and recombinant proteins | | |
| Exendin-4 | In-house (Syracuse University) | N/A |
| GEP44 | GenScript (Piscataway, NJ) | N/A |
| GEP12 | In-house (Syracuse University) | N/A |
| PYY(3-36) | In-house (Syracuse University) | N/A |
| PYY(1-36) | In-house (Syracuse University) | N/A |
| PD160170 | Tocris | 2200 |
| BIBO3304 | Tocris | 2412 |
| ^3^H-2-deoxyglucose | Perkin Elmer | NET328A250UC |
| Wortmannin | Sigma Aldrich | W1628 |
| Y1-R | antibodies-online Inc. | ABIN4888949 |
| Glucagon-Like Peptide 1 Receptor (GLP-1R) protein (His tag) | antibodies-online Inc. | ABIN3080888 |
| Neuropeptide Y Receptor Y1 (NPY1R) protein (His tag) | antibodies-online Inc. | ABIN7086253 |
| ProTide Rink amide resin | CEM Corporation | R002 |
| Triisopropylsilane | Sigma-Aldrich | 233781 |
| Trifluoroacetic acid | Sigma-Aldrich | 8.08260.2501 |
| *N,N’*-Diisopropylcarbodiimide | Sigma-Aldrich | D125407 |
| Oxyma Pure | CEM Corporation | S001 |
| Piperidine | Sigma-Aldrich | 8.22299.0500 |
| *N,N’*-Dimethylformamide | VWR (Radnor, PA) | BDH83634.400 |
| α-cyano-4-hydroxycinnamic acid | Acros Organics | 163440050 |
| Acetonitrile HiSolv | VWR | BDH83639.400 |
| Diethyl ether | VWR | BDH67003.400 |
| Dulbecco’s Modified Eagle Medium | Sigma-Aldrich | D6429 |
| Penicillin-streptomycin | ThermoFisher Scientific | 15140122 |
| Bovine serum albumin | Sigma-Aldrich | A3059 |
| Fetal bovine serum | Sigma-Aldrich | 12303C |
| Liberase | Roche Molecular Biochemicals (Indianapolis, IN) | 05339880001 |
| Euthasol, 390 mg/ml sodium pentobarbital | Virbac | RXEUTHASOL |
| Insulin | MilliporeSigma | 91077C |
| Catheter lock solution (500 USP units/ml heparin in 50% glycerol) | Instech Labs (Plymouth Meeting, PA) | USP-HGS-500-10-VBP-5 |
| DAPI-containing mounting media | VECTASHIELD Antifade Mounting Medium | H1200 |
| Lactate | MilliporeSigma | L9795 |
| Standard Extracellular Saline (SES) Solution | In-house (Upstate Medical University) | N/A |
| Normal donkey serum | Sigma Aldrich | 566460 |
| 5-thio-D-glucose | Santa Cruz Biotechnology | sc-221044A |
| Meloxicam | Midwest Veterinary Supply | N/A |
| isoflurane | Butler Schein |  |
| sulfo-Cyanine5 DBCO | Lumiprobe | 433F0 |
| Bay-K8644 | Sigma-Aldrich | B112 |
| Critical commercial assays and kits | | |
| hY2-R Binding Assay | EuroscreenFast (Gosselies, Belgium) | FAST-0321B |
| hGLP-1R Agonist-Based Internalization | Eurofins Discovery (Fremont, CA) | 86-0010P-2029AG |
| hY2-R Agonist-Based Internalization | Eurofins Discovery (Fremont, CA) | 86-0010P-2037AG |
| hGLP-1R Agonist-Based Arrestin Recruitment | Eurofins Discovery (Fremont, CA) | 86-0001P-2166AG |
| cAMP Kit | ThermoFisher | 4412182 |
| Amplex Red Glucose/Glucose Oxidase Assay Kit | ThermoFisher | A22189 |
| Lactate Oxidase from *Aerococcus viridans* | MilliporeSigma | L9795 |
| Beuthanasia-D | Schering-Plough Animal Health Corp., Union, NJ | N/A |
| Experimental models: Cell lines | | |
| HEK293 cells stably transfected to express hGLP-1R | In-house | N/A |
| HEK293 cells | American Type Culture Collection (Manassas, VA) | N/A |
| HEK293 C24 | American Type Culture Collection (Manassas, VA) | N/A |
| Experimental models: Organisms/strains | | |
| Adult male Wistar rats | Charles River Laboratories (Wilmington, MA) | 003 |
| Adult male Sprague Dawley rats | Envigo Harlan (Indianapolis, IN) | 002 |
| Adult male DIO Wistar rats | Charles River Laboratories (Wilmington, MA) | 003 |
| Software and algorithms | | |
| GraphPad PRISM | GraphPad Software | N/A |
| Stata/SE 14.2 | STATACorp LLC | N/A |
| ProData Viewer Software | JASCO J-715 spectropolarimeter | N/A |
| FlexStation 3 microplate reader | Molecular Devices | N/A |
| Imaris 8.1.2 software | Bitplane | N/A |
| HPEPDOCK Server | Huang Lab (Huazhong University of Science and Technology, Wuhan, China) | N/A |
| Other | | |
| High-Fat diet | Research Diets, Inc. (New Brunswick, NJ) | D12492 |
| BioDAQ cages | Research Diets, Inc. (New Brunswick, NJ) | E2 Electronic |
| DietMax Food Monitoring System | OmniTech Electronics, Inc. (Columbus, OH) |  |
| Normal chow | LabDiet (St. Louis, MO) | PicoLab Rodent 5053 |
| Kaolin (powdered) | Sigma Aldrich | K1512 |
| OneTouch Ultra Mini Glucometer | Lifescan (Malvern, PA) |  |
| OneTouch Ultra Glucose test strips | Lifescan (Malvern, PA) | N/A |
| Microvette® 100 K_3_ EDTA | Sarstedt | 20.1278.100 |
| Superfrost Plus slides | Fisher Scientific |  |
| RNAscope® Multiplex Fluorescent Reagent Kit v2 | ACDBio | 323100 |
| RNAscope probe Rn-NPY1-R-C1 | ACDBio | 414471 |
| RNAscope probe Rn-GLP-1R-C2 | ACDBio | 315221-C2 |
| RNAscope probe Rn-NPY2-R-C1 | ACDBio | 414481 |
| BZ-X800 microscope | Keyence |  |
| Liquid scintillation counter | Beckman | Model LS6500 |
| 26-gauge cannula | Plastics One |  |
| Spectrophotometer, plate reader, Synergy 4 | BioTek (Winooski, VT) | Model S4MLFPTA |
| Wizard 2, 5-channel gamma counter | Perkin Elmer | Model 2470-0050 |
| BaroFuse Multi-Channel perifusion system | EnTox Sciences | Model 001-08 |
| pcDNA3.1-hY2 receptor plasmid DNA | cDNA Resource Center | NPYR20TN00 |
| pcDNA3.1-hY1 receptor plasmid DNA | cDNA Resource Center | NPYR10TN00 |
| OpenSPR | Nicoya (Kitchener, ON, Canada) | N/A |
| Nitrilotriacetic acid (NTA) sensor chip | Nicoya Store (Kitchener, ON, Canada) | SEN-AU-100-10-NTA |
| Zorbax C18 column (5µm, 9.4 x 250 mm) | Agilent | 880995-202 |
